# The maintained attention assessment in patients affected by Myalgic encephalomyelitis/chronic fatigue syndrome: a reliable biomarker?

**DOI:** 10.1186/s12967-021-03153-1

**Published:** 2021-12-04

**Authors:** Iñigo Murga, Larraitz Aranburu, Pascual A. Gargiulo, Juan-Carlos Gómez-Esteban, José-Vicente Lafuente

**Affiliations:** 1 LaNCE-Neuropharm Group, Neuroscience Dep., University of the Basque Country (UPV-EHU), Leioa, Bizkaia Spain; 2 Experimental Psychology Laboratory, CONICET, Dep. Pathology, UNC, Mendoza, Argentina; 3 Neurodegenerative Disease Group. Biocruces Research Institute, Bizkaia, Spain

**Keywords:** Myalgic Encephalomyelitis/Chronic Fatigue Syndrome, Maintained attention, Toulouse-Piéron test

## Abstract

The maintained attention is the cause of great functional limitations in CFS/ME, a disease that mainly affects women in the central period of life. Cognitive function is explored using the Montreal Cognitive Assessment, the maintained attention using the Toulouse-Piéron test with which the Global Index of Attention and Perception (GIAP) is obtained, the fatigue using the visual analog scale and the perception of effort using the modified Borg scale. The final sample were 84 patients (66 women/18 men) who met diagnostic criteria (Fukuda-1994, Carruthers-2011) and 22 healthy controls (14 women/8 men). Most of patients maintain normal cognitive function, showing low or very low attention score in the 70% of patients with a marked cognitive fatigue compared to the control group (p < 0.05). There were no significant differences between genders in GIAP or fatigue for CFS/ME; however, sick women perceive cognitive effort higher than men. Deficits in sustained attention and the perception of fatigue, so effort after performing the proposed test are a sensitive and reliable indicator that allows us to substantiate a clinical suspicion and refer patients for further studies in order to confirm or rule out CFS/ME.

## Introduction

Myalgic Encephalomyelitis (ME) was the name proposed by Acheson for a group of epidemic outbreaks that took place between 1945 and 1955 in different countries [1]. It already appears as a neurological illness in the International Classification of Diseases (ICD-8) [2]. Chronic Fatigue Syndrome (CFS) was described in 1988 by the Centers for Disease Control and Prevention (CDC) [3]. This nomenclature didn’t aim to replace Myalgic Encephalomyelitis (ME) and both terms (CFS/ME) were assimilated in the first world symposium (Cambridge-1990) for this pathology [4] appearing both under the neurological heading in the ICD-11 (8E49) [5].

The pathophysiology is still unknown, remaining elusive for an etiological diagnosis. It has been attributed, in part, for lacking of specific tests. At the present, there is a large number of scientific contributions, but it is still without clarifying its pathological substrate. The nuclear symptoms in this pathology are the chronic central fatigue (> 6 months) and the post-exertional malaise with recovery time longer than 24 h. Fatigue and post-exertional malaise must not be related to organic or mental pathology, being of idiopathic origin. Although, an immune compromise, such as a viral infection, is often reported by patients at the beginning of the process a morphological or biological correlation is still undefined. CFS/ME is considered a neuro-immune-endocrine dysfunction or a post-effort neuro-immune depletion.

Studies about prevalence point to a 0.89%, but with a very wide range depending on authors and the methodology. In any case, all agree that women suffer the disease at least 1.5 to twice more higher than men [6].

In spite of the plethora of symptoms showed usually by these patients often neurological manifestations are specially relevant and among them cognitive disorders have been described playing a central role [7, 8]. A meta-analysis concluded that the cognitive deficit is a primary finding independently of emotional state of patients. The deficit depends mainly on the compromise of *attention*, memory and reaction time. There isn´t motor deficit, vocabulary, reasoning and global functioning [9].

Attention is a multidimensional phenomenon, ambiguous and elusive, a central function devotes to control and guide the conscious activity according to a specific objective [10]. The present work is carried out through a simple test, which can be used by the general practitioner in order to orientate the evaluation of patients or by researcher to evaluate the follow up.

## Material and methods

One hundred and thirteen participants were recruited, 91 affected with CFS/ME and 22 controls, during the period October 2017–December 2019.

The study was approved by the Ethics Committee for Research with Human Beings (CEISH) of the University of the Basque Country (nr. 80/2016 of 27/10/2016). All participants sign the informed consent.

The inclusion criteria were: age among 18–68 years, of caucasian ethnicity, provide a medical report for CFS/ME diagnose, a general blood-urine analysis in the last 6 months and a normal ECG-rest. All patients meet the criteria of Fukuda-1994 and/or Carruthers-2011.

The exclusion criteria were: be unable to conduct clinical sessions or filling out the research questionnaires, be participating in another study, pregnancy, lactation, major surgical intervention in the last year, treatment with chemotherapy, radiotherapy, immunotherapy or systemic steroids during the investigation period, drug abuse in the past or present, morbid obesity, a history of cranial surgery or severe head-brain trauma. Controls don´t suffer from diseases or take drugs.

This is a retrospective and case–control study. During the evolutionary course of the investigation, two patients were excluded, one for presenting a diagnosis of multiple sclerosis and the other for starting treatment with Rituximab. Additionally, two patients leave the study for impairing their illness and three don´t indicate the cause.

Participant didn´t modify their daily routine, regarding drugs intake for ethical reasons. The session were always conducted by the same physician team. Tests were performed in a silent room under standard conditions of temperature and humidity, among 17:00 and 19:00 h, without general discomfort from cold, flu-like, trauma, fever or woman do not having the period.

The first session was devoted to perform the Montreal Cognitive Assessment (MoCA) [11]. MoCA has been designed to assess mild cognitive impairment. This instrument examines following skills: attention, concentration, executive functions (including ability for abstraction), memory, language, visual-constructive capacities, calculation and orientation. The administration requires approximately five to ten minutes, before starting some instructions have to be given. The test has been validated in Spanish (Lozano et al). It has a maximum score of 30 points, a cut-off score of 13 points for dementia and 20 points for mild cognitive impairment [12].

In the second session, the maintained or executive attention is assessed by the Toulouse-Piéron test. This questionnaire was constructed to have an instrument that would allow the measurement of sustained attention—concentration and resistance to monotony. According to the bibliography [13], the proposed test is one of the most relevant for this scope. In the present study was used the eighth edition adapted to Spanish. The test contains 1.600 graphic elements (squares that have a dash on one of their sides or edges) distributed in 40 rows [14]. It is used to obtain the Global Index of Attention and Perception (GIAP). Prior to start and after answering, participant reflects their perception of fatigue in a visual analogue scale (VAS-fatigue) [15]. Finally, the modified scale of perception effort (Borg) is completed [16, 17].

### Statistical analysis

The scope of the study is to compare the behavior of variables in both groups. Therefore, with the aim of finding the most appropriate analysis in each case, normality tests have been performed, such as Kolmogorov–Smirnov or Shapiro–Wilk, depending on the sample size for each case. Once these contrasts have been carried out, the equality of means has been tested in the sample groups using parametric tests (t-student) if the studied variables could be considered normal, and U Mann–Whitney contrasts otherwise. Statistical significance was considered when p < 0.05, using bilateral contrast. Analysis have been carried out using the Studio Version 0.99.489 program and the graphics have been made with microsoft excel.

## Results

The final sample consisted of 84 CFS/ME patients, with a mean age of 44.41 ± 9.37 years (range of 18–61), being 66 female and 18 male, and 22 (14 females and 8 males) serve as controls with a mean age of 45 ± 13.15 years (range of 18–64). There were a greater number of women among the patients, almost in a ratio 4:1. The affected group corresponds mainly to the age group of 35–51 years (64.28%). For ethical reasons medication such as non-steroidal anti-inflammatories, anxiolytics and anti-depressant was not withdrawn. Only the 16.6% of the sample didn`t take any drug daily.

Educational level of patients and control participants is recorded in order to avoid out bias due to this feature. Groups showed the following distribution: with an university degree the 61% of CFS/ME patients and the 64% of controls; high school 20% and 9% respectively; technical training a 14% in both groups and primary studies the 5% of CFS/ME and the 14% of the control group.

The scores of MoCA test showed significant differences between CFS/ME-group and control-one, being higher the score for control group but both were inside the normality-rank. Only some cases of CFS/ME-group (N = 5; 3 females/2 males; 6%) presented a mild cognitive impairment (< 21 score). Women from CFS/ME group performed the test significantly better than men (Table 1).

**Table 1:** Data of the tests used in the cognitive evaluation

Variable^Test^	CFS/ME N = 84 µ(σ) range	Control N = 22 µ(σ) range	p	CFS/ME		
				Women N = 66 µ(σ) range	Men N = 18 µ(σ) range	p
Cognitive function^MoCA^	25.76 (2.45) 18–30	27 (2.33) 21–30	*	26.06 (2.26) 18–30	24.66 (2.80) 18–29	*
Global Index Attention and Perception (GIAP)^Toulouse−Piéron^	1.94 (0.80) 0–3	3.04 (0.76) 1–5	***	1.98 (0.76) 0–3	1.77 (0.91) 0–3	
Pre test- fatigue^VAS^	62.20 (17.10) 0–100	5.45 (16.43) 0–70	***	63.40 (16.76) 0–100	57.77 (17.57) 20–95	
Post test- fatigue^VAS^	77.91 (17.84) 10–100	6.36 (18.71) 0–70	***	78.63 (17.87) 10–100	75.27 (17.51) 30–100	
Perception of effort cognitive^Borg modified^	7.70 (3.01) 3–10	1.77 (0.99) 1–5	***	8.12 (2.81) 3–10	6.16 (3.23) 3–10	*

U Mann–Whitney test

t-student test (CFS/ME: women pre test- fatigue vs. men pre test- fatigue, women post test- fatigue vs. men post test- fatigue)

Cognitive function: Montreal cognitive assessment (MoCA). Cut-off point: 26. Mild Cognitive Deterioration (< 21)

Global Index Attention and Perception (GIAP): Toulouse-Piéron test. 0 = No evaluable (abandons the test due to very high fatigue), 1 = Very low, 2 = Low, 3 = Medium, 4 = High, 5 = Very high

Fatigue: Visual analogue scale (VAS). 0 mm = No fatigue, < 40 mm = Mild, ≥ 40 mm–< 70 mm = Moderate, ≥ 70 mm = Severe, 100 mm = Maximum

Perception of cognitive effort: Borg modified scale. 0 = Nothing at all, 1 = Very, very light, 2 = Very light, 3 = Moderate, 4 = Something hard, 5 = Hard, 6 = Hard, 7 = Very hard, 8 = Very hard, 9 = Very hard, 10 = Very very hard

* p < 0.05

** P < 0.01

*** P < 0.001

**Fig. 1:**
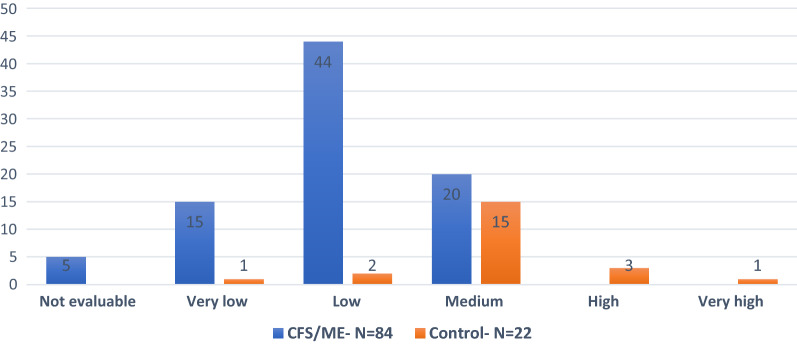
Maintained attention with Global Index of Attention and Perception (GIAP). The figure reflects the distribution of the number of patients, placed above the bars (ordinate axis), for the categorization of the Global Index Attention and Perception (GIAP). Note the cognitive deficit in maintained attention for CFS/ME in 10 min (76%- N = 64). Five sicks are unable to finish the test due to very high mental fatigue

Regarding maintained attention test, there were significant statistically differences in the Global Index of Attention and Perception (GIAP) between CFS/ME and control groups, but not among women and men with the syndrome. Attention records were mainly low (52%), very low (18%) and not evaluable (6%), this last by abandonment due to very high fatigability experimented by some sick. Regarding attention deficit only 20 patients of CFS/EM- group (24%) arrived to medium level, meanwhile in the control group was the predominant step (68% of participants). There were not statistically significant differences among gender in CFS/ME- group for any level, being most of patient in the low level (Fig. 1).

Fatigue evaluation show highly significant differences between both groups in any situation (Table 1). Comparing the evolution of this fatigability after the administration of the Toulouse-Piéron test is remarkable the highly significant differences (p < 0.001) reported by CFS/EM- group (women as well as men) stand out compared to the no difference reported by the control group (Table 2).

**Table 2:** Data of perception fatigue pre vs. post with Tolouse-Piéron test

Variable^Test^	CFS/ME N = 84 p	Control N = 22 p	CFS/ME	
			Women N = 66 p	Men N = 18 p
Pre-test fatigue vs. post-test fatigue^VAS^	***		***	***

U Mann–Whitney test (control, CFS/ME men)

t-student test (CFS/ME, CFS/ME women)

The marked and rapid fatigability in CFS/ME in 10 min, with no impact on control. There are differences in women and men with CFS/ME between pre-test fatigue vs. post-test

^*^ p < 0.05

^**^ P < 0.01

^***^ P < 0.001

Perception of effort referred by CFS/EM patients was significantly higher than for control participants (p < 0.001). In addition, comparing gender in that group women showed higher levels than men (p < 0.05; Table 1).

Some patients (N = 15) provided a psychiatric diagnosis of reactive depression. It should be noted that, there were not differences in GIAP between these patients and the rest (N = 69**;** p = 0.116).

## Discussion

Patients diagnosed of CFS/ME reported fatigue (physical and mental), according to the definition of the Japanese Society of Fatigue Science: “a decline in the ability and efficiency of mental and/or physical activities that isn´t caused by excessive mental or physical activities or disease” [18]. A medical evaluation of the studied patient didn´t reveal any alteration that could explain their general weakness or tiredness and much less a hard and rapid cognitive fatigability with stressful perceived effort referred after a demanding cognitive task for 10 min.

For instance, various studies have reflected changes referred to an increase in ventricular lactate. It may be registered by Magnetic Resonance (spectroscopy) and may be the consequence of an increased anaerobic metabolism [19–22]. Glutathione decrease points to a deregulation of oxidative metabolism and mitochondrial function, aspect to be considered in marked fatigability [21, 23, 24]. However, these changes are not specific of CFS/ME, since they have also been evidenced in other pathologies [25]. Cerebral perfusion decrease have been probed by various techniques (Magnetic Resonance-RM Arterial Spin Labeling), showing consistent results [26–28]. Fatigue mechanisms remain still unknown and some of these points are essential parameters for monitoring brain metabolism. The increase in lactate and free radicals has been referred to explain this cognitive deterioration. However, our team points to a possible inability of the glymphatic system. It would be unable to eliminate the metabolic waste, a hypothesis alluded to in other neurological processes with cognitive problems such as Alzheimer's disease [29].

Our study shows clinical changes using a simple test. This is a significant advantage comparing to physical exertion test or cardiopulmonary stress test [30]. These tests require of great efforts from patients. These patients are characterized by their lability, weakness and sometimes marked dysautonomia as in people with (v.g. orthostatic intolerance syndrome) [31]. Our results represent a reliable alternative and they are in line with recent meta-analysis, questioning the current scales and tools to evaluate fatigue and fatigue-inducing disorders [32].

The Toulouse-Piéron test explores different types of attention: arousal attention (alert/activation), focused attention (in front of some stimulus) and sustained attention (attending a stimulus for a long time). These kinds of attention compromise various neuroanatomic structures, pathways, neurotransmitters and their receptors. Results are in agreement with others cognitive tests (v.g. Stroop test), reflecting impairment in cognitive performance. The cognitive impairment points to the neural networks involved in attention and focus the pathological substrate in areas like the anterior cingulate cortex, lateral ventral prefrontal cortex, basal ganglia or locus coeruleus [33–35]. Some of them have been explored using MRI (DTI-diffusion tensor). No significant differences in volume of gray matter and other parameters have been found. Only one research group referred the increase of anisotropy in the arcuate fasciculus in a small sample of patients, postulating this feature as a potential biomarker [36]**.** However, by the time no one other group has verified these findings.

Robison et al. (2019) reported impaired autonomic control of the heart rate (dysautonomia) associated to reductions in basic processing speed [37]. On the fatigability, the neuroendocrine system plays a key role, since there is a deficit in the modulation of stress mechanisms. Several authors have published irregular changes of cortisol levels, remaining unclear its role in mental fatigue and attention deficit [38–43]. However, a hypoactive HPA axis in these patients seems to be more a consequence than a cause. It remains to be elucidated the role played by paraventricular nucleus, pituitary and adrenal glands (HPA) in the cortisol metabolism. Neurobiological studies suggest the role of the Bed Nucleus of the Stria Terminalis (BNST) as modulator of mood, anxiety and fear connecting essential emotional processing regions, such as the prefrontal cortex, the hippocampus and the amygdala with the paraventricular nucleus triggering the stimulation of the hypothalamic–pituitary–adrenal axis [44].

In general people with CFS/ME perceive aerobic exercise as more effortful than healthy adults, this aspect is reflected in a meta-analysis but the exact causes are unclear [45]. The relevance of this effect requires further studies in order to elucidate the underlying mechanisms. They could provide information about the pathophysiology of the syndrome. Furthermore, it is to be taken into account that the effort perception could be related to differences in the immune system between genders and also to changes in the hormonal profile [46]. Recent investigations in peripheral blood mononuclear cells have shown a decrease in the ability to provide adequate intracellular levels of ATP, but only in six patients [47], aspect that needs a higher level of research to know the response to effort.

The own nature of a cross-sectional study and the uninterrupted intake of medication for ethical reasons, must be taken into account as potential limitations of the present study. Wider cohorts are necessaries to find significant clustering of homogeneous phenotypes since this is a pathology with a high clinical heterogeneity [48] and multiple comorbidities.

Future studies, using advanced technologies (v.g. RM/PET-scan, magneto-electroencephalography) should deep into attention deficit, especially stratifying phenotypes or subgroups based on clinical characteristics. Brain areas involved in this function are worth of monitoring, since it has been observed that just a 10 min attentional test deteriorates it significantly.

## Conclusion

General cognition remains preserved in most patients, only a small group of them shows a significant mild cognitive impairment. Maintained attention is clearly deficient, showing a marked fatigability after the Toulouse-Piéron test. The effort was perceived as very hard by both gender, but higher by women.

This study proposes a simple clinical way to assess maintained attention. Present results support the reliability of maintained attention as biomarker of CFS/ME. Attention deficit is a significant disability in patients affected of central fatigue.

## Data Availability

Not applicable.
